# Booming far: the long-range vocal strategy of a lekking bird

**DOI:** 10.1098/rsos.170594

**Published:** 2017-08-16

**Authors:** C. Cornec, Y. Hingrat, T. Aubin, F. Rybak

**Affiliations:** 1 Université Paris-Sud, CNRS, Neuro-PSI, équipe communications acoustiques, UMR 9197, Orsay 91405, France; 2 Emirates Center for Wildlife Propagation, PO Box 47, Missour, Morocco; 3 RENECO International Wildlife Consultants, LLC, PO Box 61741, Abu Dhabi, UAE

**Keywords:** exploded lek, houbara bustard, low-frequency vocalization, male–male competition, multi-modal coding–decoding process, redundancy

## Abstract

The pressures of selection acting on transmission of information by acoustic signals are particularly high in long-distance communication networks. Males of the North African houbara bustard (*Chlamydotis undulata undulata*) produce extremely low-frequency vocalizations called ‘booms’ as a component of their courtship displays. These displays are performed on sites separated by a distance of on average 550 m, constituting exploded leks. Here, we investigate the acoustic features of booms involved in species-specific identity. We first assessed the modifications of acoustic parameters during boom transmission at long range within the natural habitat of the species, finding that the frequency content of booms was reliably transmitted up to 600 m. Additionally, by testing males' behavioural responses to playbacks of modified signals, we found that the presence of the second harmonic and the frequency modulation are the key parameters for species identification, and also that a sequence of booms elicited stronger responses than a single boom. Thus, the coding–decoding process relies on redundant and propagation-resistant features, making the booms particularly well adapted for the long-range transmission of information between males. Moreover, by experimentally disentangling the presentation of visual and acoustic signals, we showed that during the booming phase of courtship, the two sensory modalities act in synergy. The acoustic component is dominant in the context of intra-sexual competition. While the visual component is not necessary to induce agonistic response, it acts as an amplifier and reduces the time of detection of the signaller. The utilization of these adaptive strategies allows houbara males to maximize the active space of vocalizations emitted in exploded leks.

## 1. Introduction

During propagation, acoustic signals may be subject to modifications by various processes occurring along the transmission channel [1]. Consequently, the ability to communicate by acoustic signals is often limited by the distance over which the vocalization may be perceived and decoded by a conspecific. This distance corresponds to the ‘active space’ of the signal [2] defined as ‘the distance from the source over which signal amplitude remains above the detection threshold of potential receivers’ [3] and as ‘the distance over which its meaning can be transmitted’ [4]. The active space of a signal depends on its acoustic structure and on the properties of the environment (vegetation, humidity, temperature, topography, background noise, etc.) in which it is transmitted. Signal propagation through the environment leads to a reduction of the signal-to-noise ratio caused by the spherical spreading of the acoustic wave (−6 dB by doubling of distance) and by excess attenuation due to atmospheric absorption, deflection, ground effect and multiple scattering [1,5–10]. Propagation also induces the degradation of a signal's spectral, temporal and structural characteristics, caused by reverberation, irregular amplitude fluctuations from non-stationary atmospheric turbulences, and selective frequency filtering [1,5–9,11,12]. Thus, due to propagation effects, the detection of signals by conspecifics over background noise, the decoding of information and the localization of the emitter can be challenging [7,13].

To counteract these effects, the signal structure, the coding/decoding process and the behaviour of the emitter and the receiver should be adapted to maximize signal efficiency. With this in consideration, it has been hypothesized that local habitat structure plays an important role in driving signal evolution, imposing a selective pressure on the characteristics of sound transmission. The so-called ‘acoustic adaptation’ or ‘signal structure’ hypothesis [14–16] predicts that the acoustic characteristics of a sound are suited to maximize transmission in a given habitat (native habitat). Numerous studies support this concept, providing evidence of intra- and inter-species adaptations to local habitat conditions [17–19]. However, despite supporting evidence from some studies, the hypothesis has not been universally accepted [20–23]. Signal structure may be also shaped by habitat-related background noise. Many habitats possess a typical pattern of background noise (anthropogenic, abiotic and biotic noises) which may also drive the evolution of bird vocalizations [24]. This is especially true when the frequency spectra of the signal and noise overlap. In this case, individuals may use frequencies different from ambient noise [25–28]. When the signal-to-noise ratio is weak due to strong background noise or to propagation at long range, individuals may increase the sound amplitude (Lombard effect), or may use a redundant signal repeating several times the same information [29–34]. Thus, given that the information content may depend on which acoustic characteristics are most resistant to degradation and attenuation, animals may adapt their signalling strategies to propagation constraints [35–38].

The emitter's behaviour may also be adapted to environmental constraints. For example, to enhance the probability of detection of its calls by conspecifics, the signaller may gain a clear benefit from concentrating its circadian calling activity during the time of the day most efficient for signal propagation [39] or avoiding temporal overlap with other species using the same frequency band [40,41]. In addition, individual location and posture can also improve signal propagation, as shown in many songbirds where perching on elevated spots enhances their active space [42–45]. Finally, complex behavioural routines where several signals, involving several sensory modalities produced serially or simultaneously, can enhance the information transfer or can complement each other [46,47].

Additional constraints acting on the distance and consequently on the communication between individuals are social (mating behaviour) and density-dependent (resource availability). Among mating systems, some species have developed a singular spatial organization, called an exploded lek, where individuals are typically separated by large distances (in comparison with classic leks) and so require long-range communication [48,49]. In this context, signallers could, therefore, have a selective advantage if their signals can travel over long distances with minimal loss of information, and receivers may benefit from gaining the maximum information possible to properly adjust their behavioural response. As a consequence, individual fitness might be linked to the effectiveness of this long-distance communication. Thus, communication in species using an exploded lek system represents an excellent model to study signal structure adaptation, coding/decoding strategies and the other behavioural traits ensuring that the information content along transmission channels remains reliable despite the large distances between individuals.

The North African houbara bustard (*Chlamydotis undulata undulata*) possesses an exploded lek socio-sexual system [50,51]. Males compete for display sites before females start to breed and remain faithful to their sites separated by large distances—on average 550 m [51]—that they defend up to five months during the breeding season, and also between years [51]. In this context, intra-sexual competition is high and intra-sexual communication is expected to be of crucial importance for males to gather and hold a display site within the lek. The courtship is particularly conspicuous with black and white ornamental feathers erected, and mainly composed of two phases: the running phase, where the male sharply drops his head on his back and begins frantically running, and the booming phase, where the male produces a stereotyped sequence of low-frequency sounds called booms [52,53]. The booms have an average fundamental frequency of 46 Hz [52,53]. Producing low-frequency sounds is known to be an effective strategy for long-range communication [54,55], and booms have indeed be recently demonstrated to be involved in communication between houbara males within the exploded lek [56].

In this study, we investigated the different adaptations at the emitter and receiver level (signal structure, coding/decoding process and bird behaviour) occurring during the courtship of the species. We first assessed the modifications of boom signals during their propagation through the species' natural habitat. Considering the species' circadian courtship activity and that the variations of daily climatic conditions in their habitat are likely to be responsible for differential sound degradation, propagation experiments were carried out at different times of day. Then, with playback experiments on males, we investigated the acoustic parameters involved in species-specific recognition. Finally, we performed two other playback experiments in order to study the role of redundancy in the boom sequences and the potential synergetic role of visual and acoustic signals in the decoding process.

## 2. Material and methods

### 2.1. Study area

The study was carried out in 2012, 2013 and 2014 from March to April in a 663 km^2^ area called Al Baten, which extends from the Middle Atlas to the Moulouya River in eastern Morocco (33.23° N, 03.94° W). Additionally, in 2013 fieldwork was conducted between April and May in a high plain bordered by smooth relief located near Enjil, within the Middle Atlas (33.05° N, 04.35° W). Altitude ranges between 800 and 1700 m.a.s.l. in Al Baten, and is about 1600 m.a.s.l. in Enjil. These arid habitats are characterized by hills and slightly undulating gravel ‘reg’ plains, intersected by wadis and temporarily flooded areas [50]. Dense vegetation is often present in wadis, while reg is typically covered by sparse shrubby vegetation. Propagation and playback experiments were all performed in reg plains, characterized by a substrate of gravels sparsely covered by Solaneacea (*Lycium intricatum*), Chenopodiaceous plants and slightly taller bushes (such as *Launea arborescens*, *Noaea mucronata*) or graminea of height less than 45 cm [51].

### 2.2. Propagation experiment

To assess the modifications of booms during propagation, we selected one sequence of five booms from our data bank of previous recordings from 2011 and 2012 [53], and broadcast it repeatedly 10 times with a Marantz PMD671 recorder connected to a custom-built loudspeaker (DIVATECH, frequency response: 20–2000 Hz ± 6 dB) powered by a 12 V battery, at a mean intensity of 80 dB SPL (measured at 1 m from the loudspeaker with a Brüel & Kjaer 2235 sound-level meter, linear and slow setting). This value corresponds to male boom intensity measured in captivity (F. Rybak 2010, unpublished data). The repeated sequence of booms were re-recorded using a G.R.A.S. 46AE microphone (frequency response: 3.15 Hz–20 kHz ± 2 dB) positioned at 0.6 m off the ground (corresponding to the mean height of the head of a standing houbara), connected to a Marantz PMD661 recorder (sampling frequency: 44.1 kHz) over eight distances: control signal at 1 m, and propagated signals at distances of 20, 40, 80, 160, 320, 640 and 1280 m (along a one line transect). The propagation experiment was carried out in Al Baten area in April 2012 and 2013.

Considering male display activity occurring at intervals across a 24 h period, we divided each day into four distinct phases: afternoon (when males do not display), dusk (when males start their courtship) and night and dawn (when courtship activity is the most intense) [51]. Propagation experiments were then conducted under good weather conditions (wind with a velocity of more than 4 m s^−1^ and rainy days were avoided) from 14.20 to 15.38 (afternoon), from 17.33 to 18.38 (dusk), from 2.56 to 4.10 (night) and from 5.44 to 6.39 (dawn).

Boom modifications were quantified by comparing the propagated sequences with the control sequence. Of the 10 repetitions of the sequence of booms recorded for each distance, only the six of highest quality were used, and only the third boom of each was analysed. Using Avisoft-SASlab Pro (R.Specht, v. 4.40; Avisoft Bioacoustics, Berlin, Germany), modifications of the amplitude modulations were assessed by calculating smoothed envelopes (Env). Modifications of frequency composition were assessed by calculating the signal spectrum (Spe) using fast Fourier transforms (window size: 4096 data points, overlapping: 50%, bandpass: 0–15 Hz), and modifications of the frequency modulations were assessed by computing digital spectrographic (Spg) cross-correlations using Avisoft Correlator (for the method, see [57]). Each of these measures were averaged (*N* = 6) for each propagation distance.

In the study sites, climatic conditions are known to greatly vary between the four daily sample periods, and thus expected to influence sound propagation. Therefore, we collected temperature, humidity and wind speed data from 15 March to 30 April 2012 and from 1 March to 30 April 2013 from a weather station located at 45.7 km from the location of the loudspeaker, in the same river basin along the same mountain chain at 850 m.a.s.l. (Campbell scientific GRWS100).

To determine the level of the ambient noise in the species' habitat, we used a Voltcraft SL-451 sound-level measurement device (reading range 30–130 dB, accuracy ±1.4 dB, linear scale, slow setting). Ambient noise was recorded in the same location as the propagation experiment, and was recorded for each of the four time periods in 5 min (one measurement per second) samples on two different days (10 and 11 April 2014), under good weather conditions, i.e. with a wind velocity of less than 4 m s^−1^.

### 2.3. Playback experiments

Three playback experiments were conducted to assess the coding–decoding strategies. Specifically, the importance of the acoustic features the most resilient in the propagation experiment was tested using experimentally modified signals: frequency features and frequency modulation in playback experiment 1, and redundancy in playback experiment 2. Moreover, the importance of multi-modal signals was tested in playback experiment 3. Playback experiment 1 was performed in Al Baten area from 20 March to 15 April 2013 on 12 males. Playback experiment 2 was performed in Al Baten area from 5 to 31 March 2014 on 12 males. Playback experiment 3 was performed in Enjil area from 28 April to 27 May 2013 on 11 males; an additional two males were also tested in Al Baten between 3 April and 5 April 2014 to increase the sample size. Each male selected was tested in one single playback experiment. These males were observed for several days before each experiment to assess their faithfulness to their display site and to determine their preferred display location. Since 2002, display sites have been located every year in Al Baten following a standardized method [51]. A total of 66 and 70 display sites were identified in 2013 and 2014, respectively. In Enjil, as less intensive surveys were conducted prior to 2013, circular observations were carried out during the 2 h after dawn and the 2 h before dusk during two months in 2013. A total of 16 display sites were identified in this area using these methods. As not all the males were individually tagged, we cannot entirely exclude the risk of testing the same individual on two different display sites. However, surveys of males equipped with VHF transmitters conducted between 2002 and 2007 showed their high fidelity to their display site during the breeding season and across breeding seasons [58].

#### 2.3.1. Signals played back

Houbara bustard males produce sequences of booms including on average 6.44 ± 1.44 booms (range = 2–11) and lasted on average 13.46 ± 3.05 s, each boom being characterized by a very low fundamental frequency, with a mean value of 46.52 ± 2.11 Hz (range = 40–54) [53]. We used previously recorded natural sequences of booms to prepare the signals played back.

**Figure 1.**
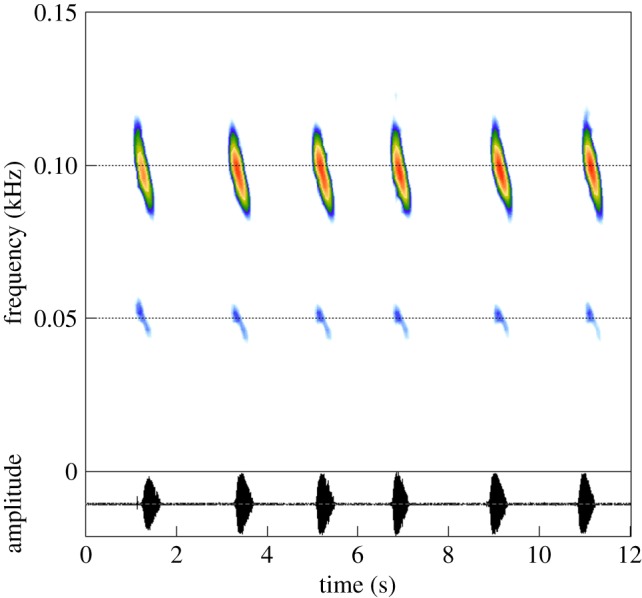
Spectrogram of a natural sequence of booms used as a control signal (C signal) in playback experiment 1.

Playback experiment 1: we tested each male with three categories of signals: a control signal corresponding to a natural sequence of six booms (C signal; see an example in figure 1), the same sequence filtered with a bandpass [80–100] Hz, resulting in a sequence of booms with only the second harmonic present (second H signal), and the same sequence of booms without frequency modulation (WFM signal), obtained by equalizing at the same level the frequencies of the booms using the Avisoft-SasLab Pro graphic synthesizer (electronic supplementary material, figure S2).

Playback experiment 2: we tested each male with three categories of signals differing by the number of booms per sequence: one single boom signal (1B), a signal consisting of the same boom repeated five times (5B) and a signal consisting of the same boom repeated 10 times (10B). To build 5B and 10B signals, we used natural sequences of 5 and 10 booms in which the original intervals of silence between booms were kept but all the different booms were replaced by the same single boom.

Playback experiment 3: each male was tested with acoustic and visual lures. The acoustic lure consisted of a natural sequence of six booms played back and the visual lure consisted of a dummy houbara male. Each lure was either presented alone, as a unimodal acoustic signal (A), as a unimodal visual signal (V) or as a multi-modal signal combining acoustic and visual signals (AV).

The sequences of booms used for these tests had been previously recorded in 2010 and 2011 (for details of recordings, see [53]) from males not tested in this study. All the booms had a high signal-to-noise ratio, with frequency and temporal parameters within the natural range of the species, and all were rescaled to match their root mean square amplitude at the same output. For each acoustic signal, the sequence of booms was repeated five times with two successive sequences separated by 3 min of silence; this corresponds to the average natural interval between sequences of booms, thus making a total duration of 13 min. To avoid pseudo-replication [59], three different sequences were prepared for each category of signal.

**Figure 2.**
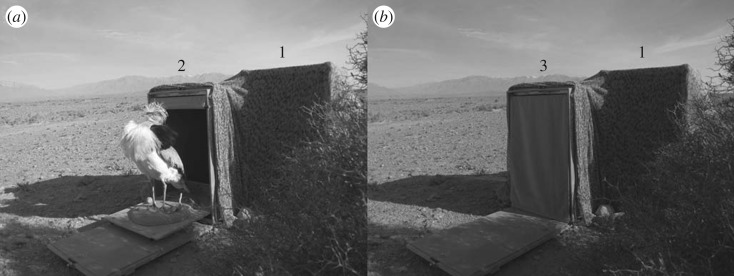
(*a*) The dummy male houbara bustard, used for the playback experiment 3, out of the box with the curtain raised (2, height = 69 cm) and the loudspeaker (1, height = 75 cm). (*b*) The dummy male inside the box with the curtain down (3).

The visual signal was a sexually mature male houbara stuffed and mounted in a booming posture, showing its conspicuous white and black feathers (figure 2). This male was a captive-born adult male of 3 years old (born in 2009, died in 2012) which died naturally (trauma) at the Emirates Center for Wildlife Propagation (Missour, Morocco). It was fully feathered and its body size was within the normal natural parameters of the species. The stuffed lure was fixed on a wood board coated by fine brown sand and mounted on rails in a wooden box (41 × 63 × 69 cm) coated with brown sand mimicking the natural ground. The box had one open side masked by a brown curtain, making the stuffed male entirely hidden when it was inside the box. The curtain and the dummy male were manually moved by an experimenter situated behind the box to make the stuffed male move outside (visible) or inside (hidden). The presentation of the visual lure followed the same pattern as the presentation of the acoustic lure: the stuffed male was made visible for a duration of 14 s (corresponding to the mean duration of a boom sequence), five times and each presentation was separated by an interval of 3 min for a total of 13 min.

#### 2.3.2. Playback procedure

All experiments were performed at dawn, from sunrise to 2 h after sunrise. Two hours before dawn, the playback set-up (see §2.2) was positioned on the ground, under a mesh net, close to the target display site (on average 175 ± 56 m). The distance between the playback set-up and the display site was a trade-off between being as close as possible and avoiding signal alteration due to obstacles, such as microtopography or vegetation. The box containing the stuffed male was placed close to the loudspeaker and positioned so its opening side pointed towards the tested male (figure 2). One observer, lain down under a mesh net close to the loudspeaker, activated the broadcast of the signals. A second observer was installed 300 m from the display site to watch and video-record the behaviour of the tested male and to give instructions to the first observer using a walkie-talkie (i.e. when to start and end the broadcast of the signals). All experiments started at dawn, when the male was on its display site. None of tested males showed any aversion to the equipment, and all had normal courtship behaviour before playback (i.e. all performed complete sequences of courtship, including slow and stereotyped walking with ornamental black and white feathers erecting, running with the neck and the head drawn back between the wings and ornamental feathers fully erected, and booming, see [53]). Between two playbacks, the observers waited until the male came back on its initial display site and returned to normal display activity. To avoid any order effect, the playback of the different signals was randomized. On each male, each playback was performed the same day whenever it was possible (for 60% of males), or for technical reasons (weather, disturbance) on two (for 35% of males) or three (for 5% of males) consecutive days.

#### 2.3.3. Responses measured

For each trial, the behaviour of the tested male was video-recorded during the whole playback session using a high-definition camera (Sony Handycam DCR-SR190) mounted on a telescope (Swarowski ATS80 HD 20–60 × 80). The response behaviour of the male was measured for a total of 23 min—i.e. during 13 min corresponding to the broadcast of the stimulus and for 10 min of silence after the broadcast.

The following responses were scored: (i) the distance to the loudspeaker, corresponding to the ratio: minimal (male approach) or maximal (male runaway) distance male--loudspeaker after the broadcast/initial distance male--loudspeaker before the broadcast, (ii) the latency to approach, corresponding to the time between the onset of the first signal played back and the time of initiating an approach towards the loudspeaker, (iii) the percentage of time spent in courtship, (iv) the total number of courtships, (v) the mean duration of the running phase measured for each courtship performed, (vi) the mean number of booms produced, (vii) the mean duration of the booming phase and (viii) the percentage of booms emitted in the direction of the loudspeaker.

### 2.4. Statistics

To assess the effects of propagation on the signals, the means of control signals were correlated with means of propagated signals using Pearson's *r* product–moment coefficient.

To assess the variations of the climatic and background noise conditions as a function of the period of the day, the distribution of each variable, temperature, humidity, wind speed and background noise were compared between the periods of the days, using Friedman tests followed by Wilcoxon signed-rank tests.

Prior to analysing the responses in the playback experiments, in order to reduce biases associated with scaling, in some cases, values were standardized as follows using Fisher *z*-transformation [60]: standard score = (raw score − mean score for the variable)/s.d.

We used a principal components analysis (PCA) based on the correlation matrix to create a composite score with the measures of responses, which are likely to be correlated [59]. Only the first principal component (PC1) had eigenvalues greater than 1 (Kaiser's criterion) and were used as scores. Only variables estimated for all males for each experiment and within each set of signals played back were included in PCAs (distance to playback set-up, latency to approach, percentage of time spent in courtship and total number of courtships). For the other variables, and for some males, the absence of data was related to strong responses to the experiment, where males approached the playback set-up and thus stopped their courtship behaviour. As all data were not normally distributed, we used non-parametric statistics (verified by a Shapiro–Wilk test). For playback experiment 1, we used Wilcoxon signed-rank tests to compare principal components scores and estimated variables not included in the PCA of the control signal with the two modified signals (second H and WFM). For playback experiments 2 and 3, variations within principal components scores and the estimated variables not included in the PCA were compared using Friedman tests. Principal components scores and variables not included in the PCA were compared two-by-two between signals using Wilcoxon signed-rank tests. All statistical tests were performed with Statistica v. 6.1 [61].

## 3. Results

### 3.1. Propagation experiments

Regardless of the time of day, the correlations between the propagated signal (envelope, spectrum and spectrogram) and the control signal decreased as the propagation distances increased (table 1). Nevertheless, the degradation of signals was stronger in the afternoon and less severe during the night and at dawn. We could not distinguish the booms from background noise at distances beyond 640 m at dusk and at distances beyond 320 m in the afternoon. The envelopes were more strongly degraded than the spectra and the spectrograms at all periods of the day. The correlations between control and propagated spectra and between control and propagated spectrograms were higher than 0.5 until 1280 m distance in the night and at dawn, and until 640 m at dusk (table 1).

**Table 1.** Pearson *r* correlation coefficients between control and propagated signals made on envelopes (Env), spectra (Spe) and spectrograms (Spg) at four different times of the day.

time	afternoon (14.20–15.38)			dusk (17.33–18.38)			night (2.56–4.10)			dawn (5.44–6.39)		
domain (m)	Env	Spe	Spg	Env	Spe	Spg	Env	Spe	Spg	Env	Spe	Spg
1/20	0.96	0.99	0.99	0.92	1.0	0.99	0.93	0.99	0.99	0.96	1.0	0.99
1/40	0.96	0.99	0.99	0.91	0.99	0.99	0.93	0.98	0.97	0.89	0.99	0.98
1/80	0.89	0.99	0.98	0.92	0.99	0.99	0.89	0.98	0.98	0.91	0.99	0.98
1/160	0.75	0.91	0.93	0.71	0.95	0.93	0.91	0.97	0.95	0.78	0.97	0.94
1/320	0.37	0.53	0.49	0.37	0.77	0.76	0.53	0.91	0.80	0.79	0.96	0.93
1/640	—	—	—	0.17	0.63	0.57	0.56	0.71	0.63	0.31	0.75	0.61
1/1280	—	—	—	—	—	—	0.03	0.54	0.60	0.12	0.70	0.62

Analyses of weather data showed that in the arid lands where houbara males display, the temperature varied significantly between the four periods of the day (Friedman's test: *N* = 216, *χ*^2^ = 555.317, *p* < 0.001), and decreased from the afternoon until dawn (table 2). A reversed pattern was obtained for the relative humidity (Friedman's test: *N* = 216, *χ*^2^ = 492.912, *p* < 0.001) with the maximal humidity level observed at dawn and a minimal value for the afternoon (table 2 and two-by-two comparison, see electronic supplementary material, table S1). Wind speed increased during the day and then dropped significantly during the night, remaining low at dawn (Friedman's test: *N* = 216, *χ*^2^ = 329.801, *p* < 0.001; table 2 and electronic supplementary material, table S1). The level of background noise was significantly lower at night and higher at dawn and during the afternoon (Friedman's test: *N* = 602, *χ*^2^ = 797.26, *p* < 0.001; table 2 and two-by-two comparisons, see electronic supplementary material, table S1).

**Table 2.** Mean ± s.d. values of meteorological measurements and of background noise collected at four different times within 24 h. Values that share similar superscript letters did not differ significantly (Wilcoxon signed-rank tests, *p *> 0.05; electronic supplementary material, table S1). Background noise was recorded in the same place as where the propagation experiment was completed.

24 h period	temperature (°C)	humidity (%)	wind speed (m s^−1^)	background noise (dB)
afternoon	21.4 ± 4.7	25.2 ± 14.2	4. 9 ± 2.8	58 ± 8.2^b^
dusk	20.3 ± 5	31.1 ± 18.5	5.5 ± 2.5	53.9 ± 4.1
night	10.7 ± 3.3	60.4 ± 16.9	2.4 ± 2.2^a^	48.7 ± 4.2
dawn	9.8 ± 3.2	63.1 ± 16. 9	2.3 ± 2^a^	58.4 ± 5.3^b^

### 3.2. Playback experiments

**Figure 3.**
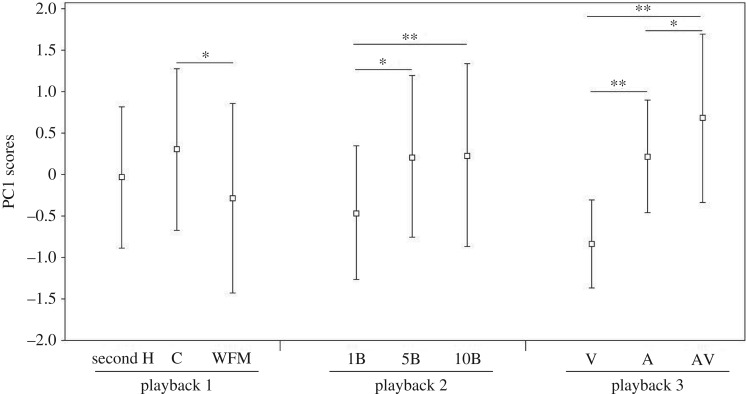
PC1 scores (mean ± s.e.) obtained for the three playback experiments. For playback experiment 1: C, control signal; second H, signal with only the second harmonic kept; WFM, signal without frequency modulation. For playback experiment 2: 1B, 1 boom; 5B, 5 booms; 10B, 10 booms. For playback experiment 3: V, visual signal; A, acoustic signal; AV, multi-modal signal (non-parametric Wilcoxon signed-rank test, *p < 0.05, **p ≤ 0.005).

Playback experiment 1. One out of 12 males did not exhibit responses to any of the three signals and was excluded from the analysis. The PC1 explained 68% of the total variance for the measured responses. Examination of the component loadings (table 3) revealed that all four responses taken into account loaded highly on PC1. As males stopped their courtship when they approached, higher positive PC1 score values corresponded to a short latency time to approach, a close approach to the loudspeaker and consequently to a decrease of the courtship activity (per cent of time spent in courtship and total number of courtships). Comparison of PC1 scores showed that males responded less strongly to the sequence of booms without frequency modulation (WFM signal) than to the control signal (figure 3 and table 4). Male responses were not significantly different between the control signal and the sequence of booms with only the second harmonic present (second H signal; figure 3 and table 4).

**Table 3.** Variance explained and loadings of the response measures on the first (PC1) principal component for the three playback experiments.

	playback 1	playback 2	playback 3
distance to the playback set-up	−0.89	−0.95	−0.93
latency to approach	−0.79	−0.81	−0.79
per cent of time spent in courtship	−0.83	−0.91	−0.85
total number of courtships	−0.80	−0.90	−0.92
eigenvalue	2.73	3.21	3.05
per cent of variance	0.68	0.80	0.76

**Table 4.** Results of non-parametric Wilcoxon signed-rank tests applied on principal component obtained for the three playback experiments. Significant results are in bold.

stimuli	*N*	*Z*	*p*-value
playback 1			
C/second H	11	1.6	0.109
C/ WFM	11	**2**.**489**	**0**.**013**
playback 2			
1B/5B	11	**2**.**489**	**0**.**013**
1B/10B	11	**2**.**578**	**0**.**001**
5B/10B	11	0.533	0.594
playback 3			
V/A	12	**2**.**824**	**0**.**005**
V/AV	12	**2**.**98**	**0**.**003**
A/AV	12	**2**.**118**	**0**.**035**

When examining the four variables not included in the PCA (Wilcoxon signed-rank tests), no significant differences between signals were found (electronic supplementary material, tables S2 and S4).

Playback experiment 2. One out of 12 males did not exhibit response to any of the three signals and was excluded from the analysis. The PC1 explained 80% of the total variance in the four responses measured (table 3). All the variables load highly on PC1. Higher positive values of PC1 scores corresponded to a fast and strong response (low latency to approach and close distance to the loudspeaker), leading to a decrease in courtship activity. Comparison of PC1 scores showed significant differences between the signals played back (Friedman's test: *N* = 11, *χ*^2^ = 11.45, *p* = 0.003). When we analysed each signal separately, PC1 scores of 5B and 10B were both significantly higher than the one of 1B (figure 3 and table 4). No significant difference was found between PC1 scores of 5B and 10B (figure 3 and table 4).

Friedman's tests performed on response variables not included in the PCA revealed that the percentages of booms emitted toward the playback set-up were significantly different between the signals (*N* = 7, *χ*^2^ = 7.714, *p* = 0.021; electronic supplementary material, table S3). A significant difference was obtained between 5B and 1B signals (1B signal: 0.164 ± 0.218, 5B signal: 0.491 ± 0.296, Wilcoxon signed-rank tests: *N* = 9, *Z* = 2.665, *p* = 0.007; electronic supplementary material, tables S2 and S5). A difference close to the significance was also obtained between 10B signal and 1B signal (10B signal: 0.455 ± 0.366, Wilcoxon signed-rank tests: *Z* = 1.836, *N* = 10, *p* = 0.066; electronic supplementary material, tables S2 and S5). For all other variables, no difference was found between signals.

Playback experiment 3. One out of 13 males did not exhibit response to any of the three signals and was excluded from the analysis. The PC1 explained 76% of the total variance measured (table 3). The four variables loaded highly on PC1. Comparison of PC1 scores showed significant differences between the signals (Friedman's test: *N* = 12, *χ*^2^ = 15.167, *p* < 0.001). Two-by-two comparisons revealed that the PC1 scores were also significantly different between the presentation of visual signal, acoustic signal and multi-modal signal. Males responded significantly more strongly to the multi-modal signal compared to the unimodal signals (figure 3 and table 4). Comparisons between the unimodal signals revealed a significantly higher PC1 score for the acoustic signal than for the visual signal. With this last signal, few approaches towards the playback set-up were observed.

When examining the four variables not included in the PCA (Friedman's test and Wilcoxon signed-rank tests), no significant differences between signals were found (electronic supplementary material, tables S2 and S6).

## 4. Discussion

Physical and social components of the environment strongly influence the communication processes. Here, we investigated the efficiency of the communication signals emitted by males of the North African houbara bustard during their courtship in spite of constraints imposed by social and environmental characteristics of their environment.

### 4.1. A long-range propagation

Propagation experiments showed that, in optimal conditions (i.e. during the night and in the absence of wind), booms were reliably transmitted up to 640 m, considering the three acoustic domains (time, intensity and frequency). However, this distance reduced during the day, dropping to 160 m in the afternoon and at dusk and 320 m at dawn. According to the correlation results obtained in the three domains, frequency modulations and spectral content were the parameters with the most resistance to propagation, and remain significantly distinguishable up to 1280 m distance in the night and at dawn, and up to 640 m at dusk. Considering that males perform their courtship at dawn, dusk and during the night, and that courtship sites are separated from each other by on average 550 ± 84 m [51], booms produced during the main periods of courtship can thus be effectively perceived by the adjacent neighbour receivers.

### 4.2. A low-frequency sound

In the context of an exploded lek, booms must be transmitted over long distance with a minimal loss of information. In the houbara bustard, booms are low-frequency signals and thus appear well adapted to communicate at long range with little attenuation and degradation compared with higher frequency sounds [1,8,54,62]. Nevertheless, propagation of low-frequency sounds may be limited for animals vocalizing near to the ground, especially in open habitats [45,63]. Indeed, ground attenuation has a notable effect on sound above 3 kHz and below 1 kHz (acoustic window [2,13,64]), and is particularly significant at less than 1 m from the surface [8]. The preferential selection by males of courtship site on elevated points with low vegetation cover and height [51] may minimize this ground attenuation effect.

### 4.3. A circadian calling activity

Animals will benefit by concentrating their acoustic signals at times of day and under weather conditions that permit the most effective long-range communication (transmission hypothesis [1,6,39,64]). Even though low-frequency sounds (like booms) are less affected by atmospheric absorption than high-frequency sounds [65,66], they remain subject to alteration by atmospheric turbulences associated with high temperature and wind. These conditions are particularly pronounced in open habitats [1,6] and play an important role in moment-to-moment variations of the excess attenuation [67]. In houbara bustard habitat, the highest temperatures, the lowest humidity levels and the strongest winds were recorded in the afternoon and at dusk. At these times of the day, the propagation of the booms is thus the worst. The courtship activity of houbara bustard males follows a circadian rhythm where booms are produced most intensively during the time of the day most effective for long-range communication. Indeed, male houbara bustards show crepuscular (morning and evening) and nocturnal display patterns with a maximal number of booms emitted during night and at dawn. This behavioural strategy allows males to minimize the alteration of their booms.

Signal propagation can also be profoundly modulated by the level of the background noise. Anthropogenic, environmental (wind) and biological (vocalizations from other species) noises can greatly affect the transmission of a sound [8]. Our recordings showed that background noise was the lowest during the night but increased from dawn to the afternoon, where it reached its maximum level. In our open habitat, environmental noise is often caused by the wind which is most intense at dusk and in the afternoon. Such environmental noise is usually of low frequency with the main part of the energy situated below 2 kHz [8] and so potentially matching with the frequency of houbara booms. However, houbara males avoid vocalizing during these periods of peak wind strength. The high level of background noise observed at dawn can be partly explained by the ‘dawn chorus’ where the song activity of many bird species is at its most intensive. This noise is dominated by high frequencies and does not have a real masking effect on low-frequency booms of houbara.

### 4.4. An adapted coding/decoding process

Despite acoustic and behavioural adaptations, some acoustic parameters degrade faster than others during propagation over long distances [1]. Therefore, the information is often encoded upon parameters possessing the most resistance to degradation and attenuation [3,37,38,68–72]. For example, in the white-browed warbler (*Basileuterus leucoblepharus*) the species identity relies on the frequency modulation, highly resistant to the propagation, whereas the individual identity and motivation are encoded on other acoustic parameters rapidly altered by propagation (public and privacy information [73]). In the blackcap (*Sylvia atricapilla*), the frequency modulation, which is resistant to propagation, is a key parameter coding species-specific information [71]. In the houbara bustard, the second harmonic and its frequency modulation appeared also to be parts of the boom which are transmitted at long range (electronic supplementary material, figure S1), and these two acoustic parameters were necessary and sufficient to elicit a territorial response of males. Thus, in houbara bustard males, the coding–decoding of species-specific information relies on stereotyped and resistant features: the decreasing frequency modulation, varying slowly up and down, and the second harmonic which contains the maximum energy [53]. This coding–decoding process remains effective over long range and may facilitate the maximization of communication distance within the intra- and the inter-sexual networks.

### 4.5. A redundant signal

According to the mathematical theory of communication [74], signallers can increase the efficiency of information transfer by increasing the duration of their signals by a redundant process. Vocalizations of male houbara bustard appear to be highly redundant with a repetition of the same information many times within the boom sequence. The results obtained in the second playback experiment showed that the broadcast of only one boom, while containing the whole information content, induced less strong responses than the broadcast of 5 or 10 booms. Thus, to ensure a reliable recognition, a series of booms is necessary to secure the transmission of the information content. Moreover, in noisy conditions, as in our case where wind generates a high level of background noise, redundancy may enhance the probability of communicating within noiseless short time-windows, as mentioned in the king penguin (*Aptenodytes patagonicus*) [31]. Sequential redundancy in animal signals is widespread [10] and has been shown in many bird species including the Eurasian chaffinch (*Fringilla coelebs*) [34] and the Japanese quail (*Coturnix coturnix japonica*) [75].

Interestingly, our results also showed that the sequences of repeated booms appeared to be more locatable than the single boom because males vocalized more significantly in the direction of the loudspeaker with sequences of 5 and 10 booms. Similar results have been found in colonies of king penguins where a high number of syllables in the signal of parents facilitate their localization by their chicks [76]. No significant difference was found between the sequences containing 5 and 10 booms, but in the wild males produce on average 6.44 ± 1.44 booms per sequence [53] which seems to be the optimum signal redundancy securing the transfer of the information.

### 4.6. A multi-modal courtship signal

In many species of birds, males produce multi-modal signals within a single display, such as plumage ornamentation, song and courtship behaviour [77–80]. According to Møller & Pomiankowski [81], elaborate multi-modal signals seem more common in polygynous species where sexual selection is intense. This is particularly true for lekking species where females evaluate several males before copulation and where males compete for lek attendance and mate access. Both males and females might benefit significantly from evaluating several cues simultaneously. Intra-sexual (competition) and inter-sexual (mate choice) processes might shape the evolution of complex multi-modal communication. Multi-modal signals may interact as a functional unit and enhance the signal efficacy and the information transfer across the communication channel [46,47,82]. Houbara bustard males perform spectacular courtship displays during the breeding season which combine visual and acoustic displays to form a composite signal. In our experiment, we examined the respective role of visual and acoustic signals and their synergetic effect during the booming phase. The results revealed a gradually increasing response of males from the unimodal visual to the multi-modal signal. Interestingly, when they are broadcasted independently, visual and acoustic signals generated different behavioural responses. In response to the visual signal alone, males remained at a distance from the stuffed male. Conversely, the acoustic signal presented alone elicits a strong agonistic response and thus appears as necessary and sufficient. In this case, males stop their courtship and approach towards the playback set-up. However, the efficacy of a dominant signal can be enhanced by its interaction with a second signal (inter-signal interaction hypothesis [47]), as shown in the squirrel treefrog (*Hyla squirella*)*,* and the túngara frog (*Physalaemus pustulosus*) [83–85]. In our case, the composite multi-modal signal induces a change in the male response compared with the isolated signals and elicits significantly higher levels of agonistic response. Territorial males approach significantly faster and closer to the playback set-up compared with the isolated signals. Here visual signal may play an important role in the timing and degree of agonistic responses, when males interact at large distances. For example, the sources of low-frequency sounds are hard to precisely locate, and visual signals may, therefore, facilitate a boom's localization and thus enhance the efficiency of the courtship display (increased detection and amplifiers [46,47]). Visual signals might also play a role in close interactions, and the movements during courtships that were not undertaken to be tested as visual signals here in our experiments might also be important.

## 5. Conclusion

The communication system of houbara bustard males seems reliable in an intra-sexual network, allowing long-range communication. The efficiency of the communication system relies on adaptive strategies occurring at different levels across the communication channel: (i) at the signal structure level with the emission of low-frequency and redundant booms enhancing the signal's active space and with the use of acoustic and visual signals acting in synergy, (ii) at the bird's behaviour level with the use of particular courtship sites and adapted time windows of sound emission and (iii) at the level of the acoustic coding/decoding process, with a species-specific recognition relying on the parameters most resistant to propagation. The exploded lek mating system represents an ideal framework for further research into the respective influence of environmental and social constraints on the evolution of communication systems.

## Supplementary Material

Table S1-S6 and Figure S1-S2 from Booming far: the long-range vocal strategy of a lekking bird

## Data accessibility

All data are available at http://dx.doi.org/10.5061/dryad.rd24d/2 [86].
