# Supplementary material for: Booming far: the long-range vocal strategy of a lekking bird
Source: R Soc Open Sci. 2017 Aug 16;4(8):170594. doi: 10.1098/rsos.170594 (PMC5579117; doi:10.1098/rsos.170594)
Supplement: Table S1-S6 and Figure S1-S2 from Booming far: the long-range vocal strategy of a lekking bird [file rsos170594supp1.docx]

**Booming far: the long-range vocal strategy of a lekking bird**

*Cornec C.^1,2^ , Hingrat Y.^3^, Aubin T. ^1^ and Rybak F.^1^

^1^ Université Paris-Sud, CNRS, Neuro-PSI, équipe communications acoustiques, UMR 9197, Orsay, F-91405, France

² Emirates Center for Wildlife Propagation, PoBox 47, Missour, Morocco

^3^ RENECO International Wildlife Consultants, LLC, PoBox 61741, Abu Dhabi, United Arab Emirates

^*^ Corresponding author: [clement.cornec@hotmail.fr](mailto:clement.cornec@hotmail.fr)

**Supporting Information**

**Table S1** Results of non-parametric Wilcoxon signed-ranks tests comparing Temperature, Humidity, Wind speed and Background noise measured at 4 different times of day

| 24h period | Temperature (°C) | | Humidity (%) | | Wind speed (m.s^-1^) | | Background noise (dB) | |
| --- | --- | --- | --- | --- | --- | --- | --- | --- |
|  | Z | **p value** | Z | **p value** | Z | **p value** | Z | **p value** |
| Night/ Dawn | 10.735 | < 0.001 | 7.082 | < 0.001 | 0.929 | 0.353 | 21.235 | < 0.001 |
| Night/ Afternoon | 12.743 | < 0.001 | 12.580 | < 0.001 | 10.688 | < 0.001 | 16.664 | < 0.001 |
| Night/ Dusk | 12.701 | < 0.001 | 12.069 | < 0.001 | 11.292 | < 0.001 | 18.959 | < 0.001 |
| Dawn/ Afternoon | 12.743 | < 0.001 | 12.678 | < 0.001 | 11.185 | < 0.001 | 1.075 | 0.282 |
| Dawn/ Dusk | 12.716 | < 0.001 | 12.231 | < 0.001 | 11.756 | < 0.001 | 16.597 | < 0.001 |
| Afternoon/ Dusk | 6.958 | < 0.001 | 8.979 | < 0.001 | 5.414 | < 0.001 | 10.459 | < 0.001 |

**Table S2** mean ± SD (N) of the measures of response obtained during the three playback experiments and for all signals played back

| **Behavioural responses** | **Playback 1** | | | **Playback 2** | | | **Playback 3** | | |
| --- | --- | --- | --- | --- | --- | --- | --- | --- | --- |
|  | **C** | **2^nd^ H** | **WFM** | **1B** | **5B** | **10B** | **V** | **A** | **AV** |
| Distance to the playback set-up | 0.44 ± 0.32 (11) | 0.53 ± 0.34 (11) | 0.88 ± 0.58 (11) | 0.87 ± 0.3 (11) | 0.62 ± 0.39 (11) | 0.55 ± 0.4 (11) | 0.99 ± 0.15 (12) | 0.57 ± 0.28 (12) | 0.38 ± 0.28 (12) |
| Latency to approach (s) | 548.7 ± 470.6 (11) | 566.87 ± 493.62 (11) | 973.91 ± 582.72 (11) | 1127.73 ± 441.72 (11) | 623.33 ± 610.6 (11) | 574.18 ± 526.5 (11) | 1288.33 ± 339.78 (12) | 544.72 ± 518.18 (12) | 251.43 ± 231.97 (12) |
| % time spent in courtship | 59.87 ± 39.48 (11) | 74.48 ± 30.69 (11) | 71.14 ± 38.83 (11) | 87.58 ± 24.56 (11) | 70.63 ± 33.22 (11) | 68.67 ± 39.97 (11) | 89.1 ± 16.56 (12) | 78.85 ± 29.05 (12) | 51.64 ± 41.41 (12) |
| Total number of courtship | 3.64 ± 4.08 (11) | 5.73 ± 3.61 (11) | 5.64 ± 4.61 (11) | 7.36 ± 3.08 (11) | 5.18 ± 3.66 (11) | 5.09 ± 4.01 (11) | 9.92 ± 3.32 (12) | 5.83 ± 2.29 (12) | 4.25 ± 3.91 (12) |
| Duration of running phase (s) | 20.83 ± 7.77 (9) | 20.9 ± 7.53 (10) | 19.36 ± 6.54 (8) | 20.48 ± 3.65 (9) | 21.26 ± 4.18 (9) | 27.96 ± 17.98 (7) | 19.28 ± 4.21 (12) | 21.26 ± 5.31 (10) | 19.69 ± 6.37 (8) |
| Number of booms | 2.9 ± 1.82 (9) | 2.72 ± 1.43 (9) | 3.63 ± 1.89 (9) | 4.15 ± 1.68 (11) | 3.69 ± 1.97 (9) | 4.19 ± 2.32 (9) | 4.2 ± 0.92 (12) | 3.17 ± 1.9 (11) | 3.66 ± 1.9 (10) |
| Duration booming phase (s) | 4.72 ± 4 (9) | 4.75 ± 3.2 (9) | 6.89 ± 4.51 (9) | 9.16 ± 3.98 (11) | 7.47 ± 4.53 (9) | 8.58 ± 5.56 (9) | 8.67 ± 2.16 (12) | 6.45 ± 4.85 (11) | 7.48 ± 4.16 (10) |
| % booms toward playback set-up | 0.55 ± 0.37 (9) | 0.66 ± 0.24 (9) | 0.38 ± 0.33 (9) | 0.16 ± 0.22 (11) | 0.49 ± 0.3 (9) | 0.46 ± 0.37 (9) | 0.36 ± 0.33 (12) | 0.46 ± 0.37 (11) | 0.41 ± 0.33 (10) |

**Table S3** Results of non-parametric Friedman tests made on 4 behavioural responses obtained during the playback experiments 2 and 3. Significant results are in bold

| **Behaviour response** | **Playback 2** | | | **Playback 3** | | |
| --- | --- | --- | --- | --- | --- | --- |
|  | N | Chi-square | p value | N | Chi-square | p value |
| Duration running phase | 6 | 2.333 | 0.311 | 8 | 1 | 0.606 |
| Number of booms | 7 | 0.286 | 0.867 | 10 | 5 | 0.082 |
| Duration booming phase | 7 | 0.286 | 0.867 | 10 | 3.2 | 0.202 |
| % booms toward playback set-up | **7** | **7.714** | **0.021** | 10 | 4 | 0.135 |

**Table S4** Results of non-parametric Wilcoxon signed-ranks tests made on 4 behavioural responses obtained on 12 males tested with a control corresponding in a natural sequence of boom (C) and two experimental signals: one signal where only the second harmonic was kept (2^nd^H) and one signal without frequency modulation (WFM). Significant results are in bold

| **Behavioural responses** | **C/2^nd^ H** | | | **C/** **WFM** | | |
| --- | --- | --- | --- | --- | --- | --- |
|  | **N** | **Z** | **p value** | **N** | **Z** | **p value** |
| Duration running phase | 9 | 0.77 | 0.441 | 7 | 1.352 | 0.176 |
| Number of booms | 9 | 0.56 | 0.575 | 8 | 0.943 | 0.345 |
| Duration booming phase | 9 | 0.14 | 0.889 | 8 | 0.845 | 0.398 |
| % booms towards playback set-up | 9 | 1.12 | 0.263 | 8 | 0.169 | 0.866 |

**Table S5** Results of Wilcoxon signed-ranks tests made on behavioural responses obtained for the three experimental signals. 1B: a single boom played back, 5B and 10B: the same boom repeated respectively 5 and 10 times. Significant results are in bold

| **Behavioural responses** | **1B/5B** | | | **1B/10B** | | | **5B/10B** | | |
| --- | --- | --- | --- | --- | --- | --- | --- | --- | --- |
|  | **N** | **Z** | **p value** | **N** | **Z** | **p value** | **N** | **Z** | **p value** |
| Duration running phase | 8 | 0.7 | 0.484 | 6 | 1.572 | 0.116 | 6 | 1.363 | 0.173 |
| Number of booms | 9 | 0.77 | 0.441 | 9 | 0.178 | 0.859 | 7 | 0.507 | 0.612 |
| Duration booming phase | 9 | 1.214 | 0.213 | 9 | 0.533 | 0.594 | 7 | 0.676 | 0.499 |
| % booms towards playback set-up | 9 | **2.665** | **0.007** | 9 | 1.836 | 0.066 | 7 | 0.338 | 0.735 |

**Table S6** Results of Wilcoxon signed rank tests made on behavioural responses obtained for the three experimental signals: for the unimodal signal (V and A) and for the multimodal signal (AV). Significant results are in bold

| **Behavioural responses** | **V/A** | | | **V/AV** | | | **A/AV** | | |
| --- | --- | --- | --- | --- | --- | --- | --- | --- | --- |
|  | **N** | **Z** | **p value** | **N** | **Z** | **p value** | **N** | **Z** | **p value** |
| Duration running phase | 10 | 1.172 | 0.241 | 8 | 0.280 | 0.779 | 8 | 0.000 | 1.000 |
| Number of booms | 11 | 1.956 | 0.051 | 10 | 1.172 | 0.241 | 10 | 1.121 | 0.262 |
| Duration booming phase | 11 | 1.422 | 0.155 | 10 | 1.07 | 0.284 | 10 | 1.07 | 0.284 |
| % booms towards playback set-up | 11 | 0.667 | 0.505 | 10 | 0.866 | 0.386 | 10 | 0.98 | 0.327 |


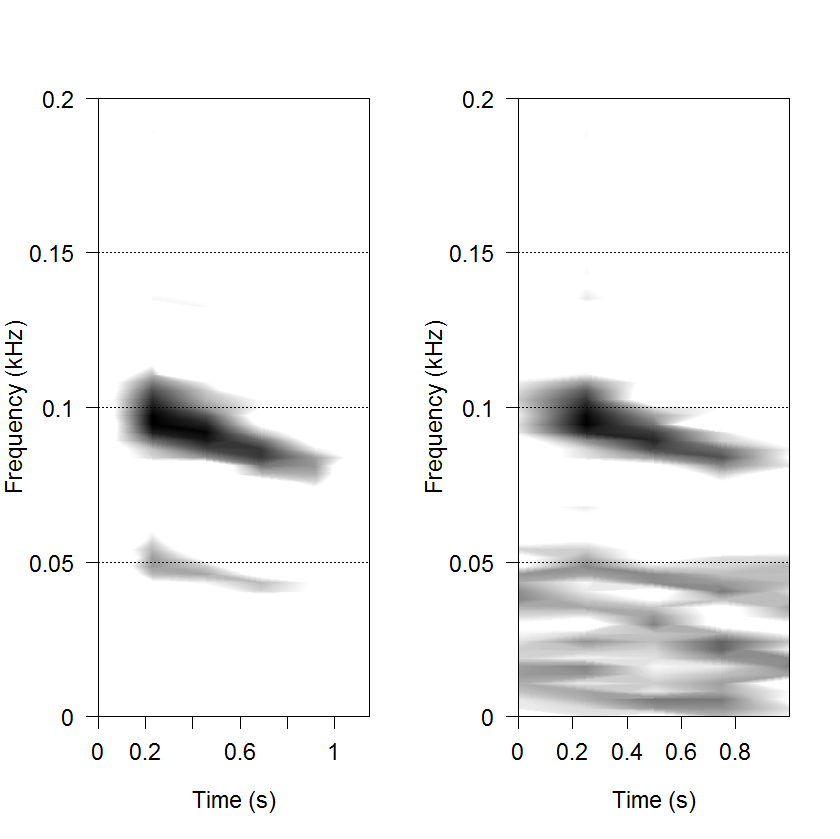


**Figure S1** Spectrograms of a natural boom emitted and re-recorded at 1 m (a) and 160 m (b) during dusk


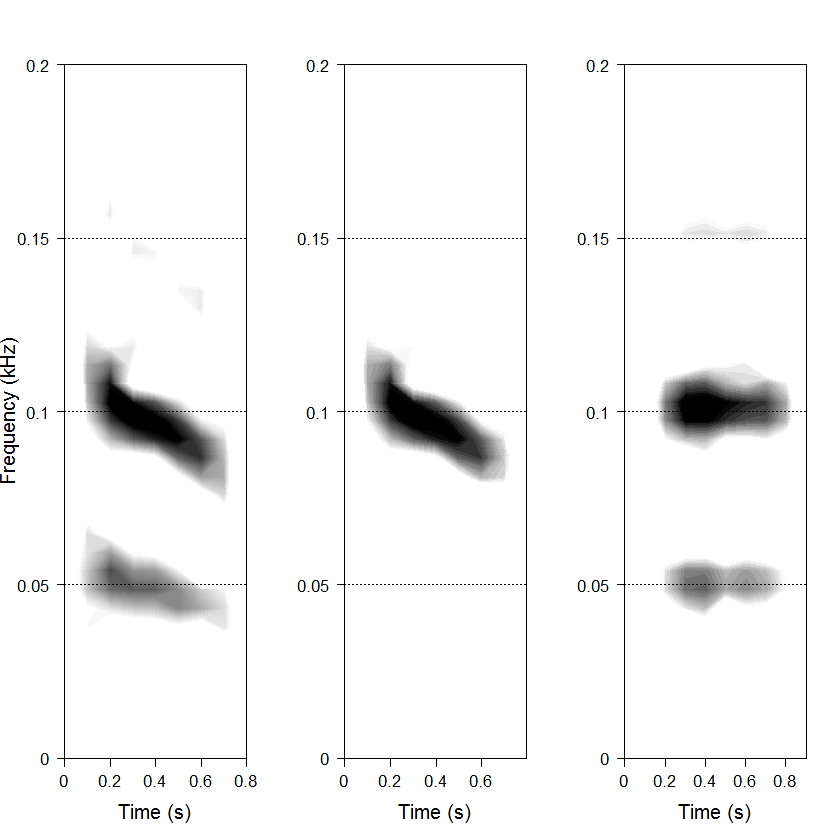


**Figure S2** Spectrograms of three booms extracted from the experimental signals used for the playback experiment 1: (a) natural unmodified boom (C signal), (b) same boom with only the second harmonic kept (2nd H signal) and (c) same boom without frequency modulation from (WFM signal)
